# Operations for Hard Cataract—Results of Extraction and of Depression or Reclination

**Published:** 1863-09

**Authors:** 


OPERATIONS FOR HARD CATARACT—RESULTS OF EXTRACTION AND OF DEPRESSION OR RECLINATION.—There are four compilations relative to the results of operations upon hard cataract. Their conclusions do not exactly correspond, although they do not vary very widely. By adding them together their errors may balance, and the result be a close approximation to the truth.

| | By Extraction. | Failures. | Ratio. | Reclination. | Failures. | Ratio. |
|---|---|---|---|---|---|---|
| Frederick Jaeger, - | 728 | 33 | 1 in 22 | 129 | 21 | 1 in 6 |
| Edward Jaeger, - - | 114 | 7 | 1 in 16 | 81 | 12 | 1 in 7 |
| Arit, - - - | 540 | 41 | 1 in 13 | 82 | 14 | 1 in 6 |
| Rivaud-Lanraud - | 2073 | 201 | 1 in 10 | 177 | 50 | 1 in 3½ |
| Total, - - - | 3455 | 281 | 1 in 12 | 469 | 97 | 1 in 5 |

Stated in another form, the number of failures after extraction of hard cataract is eight per cent., while of reclination or couching, the number of failures is twenty-one per cent. By failures are meant cases where sight was totally lost. After extraction the pupil may be closed, and, for the time, sight not be restored; an iridectomy may afterwards impart vision. Such cases are not counted failures. In reclination the immediate result may be successful, while after a few months chronic choroiditis or retinitis may be set up by the presence of the lens in the vitreous humor, and eventuate in softening and atrophy of the globe. The failures in reclination may be more or less remote, those of extraction follow immediately. The successes of reclination are liable to a disastrous issue years after the operation; the successes of extraction are permanent.—H, D. N.

